# Antioxidant, Anti-Alzheimer’s, anticancer, and cytotoxic properties of peanut oil: *in vitro*, in silico, and GC-MS analysis

**DOI:** 10.3389/fchem.2024.1487084

**Published:** 2024-10-24

**Authors:** Hanène Djeghim, Djamila Benouchenne, El Hassen Mokrani, Huda Alsaeedi, David Cornu, Mikhael Bechelany, Ahmed Barhoum

**Affiliations:** ^1^ Biochemistry Laboratory, Biotechnology Research Center (CRBt), Constantine, Algeria; ^2^ Laboratoire de Génétique, Biochimie et Biotechnologie végétale, Faculté des Sciences de la Nature et de la vie, Université des Frères Mentouri Constantine 1, Constantine, Algeria; ^3^ Higher National School of Biotechnology Taoufik KHAZNADAR, nouveau Pôle universitaire Ali Mendjli, Constantine, Algeria; ^4^ Laboratory of Applied Biochemistry, Department of Biochemistry and Cellular and Molecular Biology, Faculty of Natural and Life Sciences, University Mentouri Brothers, Constantine, Algeria; ^5^ Department of Chemistry, College of Science, King Saud University, Riyadh, Saudi Arabia; ^6^ Institut Européen des Membranes, University Montpellier, ENSCM, CNRS, Montpellier, France; ^7^ Functional Materials Group, Gulf University for Science and Technology, Mubarak Al-Abdullah, Kuwait; ^8^ NanoStruc Research Group, Chemistry Department, Faculty of Science, Helwan University, Cairo, Egypt

**Keywords:** peanut oil, phytochemical profileing, antioxidant properties, anti-Alzheimer’s potential, GC-MS analysis, enzyme inhibition, docking studies, cytotoxicity assessments

## Abstract

**Introduction:**

Peanut oil is valued for its mild flavor, rich phytochemical content, therapeutic potential, and associated health benefits. This study aims to analyze the chemical composition, antioxidant properties, and anti-Alzheimer’s potential of Algerian peanut oil using both experimental and computational approaches. The goal is to evaluate its suitability for pharmaceutical applications, particularly for its antioxidant, anti-Alzheimer, and anticancer properties.

**Methods:**

The chemical composition of the peanut oil was determined using Gas Chromatography-Mass Spectrometry (GC-MS). Antioxidant activity was assessed through DPPH and CUPRAC assays, while enzyme inhibition was evaluated using butyrylcholinesterase (BChE) inhibition assays. In silico molecular docking studies were conducted to predict interactions between key compounds and BChE. Additionally, physicochemical properties were evaluated using Lipinski’s rule of five, and cytotoxicity was tested against various cancer cell lines, including melanoma (A2058 and SK-MEL-1), non-small cell lung cancer (NCI-H838), and leukemia (H9).

**Results:**

GC-MS identified 20 chemical compounds in the peanut oil, with oleic acid as the predominant compound (41.98%). The antioxidant activity showed an IC50 value of 265.96 ± 14.85 μg/mL in the CUPRAC assay. BChE inhibition was moderate, with 36.47% ± 3.71% enzyme inhibition at 200 μg/mL. Molecular docking studies highlighted 6-methyl octahydro-coumarin with a docking score of −15.86 kJ/mol against BChE, although it was less potent than Galantamine (−23.4 kJ/mol). Physicochemical analysis revealed that oleic acid and palmitic acid exhibit logP values of 5.71 and 5.20, respectively, indicating drug-like potential. Cytotoxicity assessments demonstrated that oleic acid, palmitic acid, and stearic acid were effective against melanoma and lung cancer cells, while oxiraneoctanoic acid, 3-octyl, showed significant activity against leukemia cells.

**Discussion and conclusion:**

The results demonstrate that peanut oil possesses notable antioxidant, anti-Alzheimer, and anticancer properties. The high concentration of oleic acid, alongside moderate butyrylcholinesterase (BChE) inhibition and strong cytotoxic effects on various cancer cell lines, highlights its potential as a therapeutic agent. While 6-methyl octahydro-coumarin exhibited favorable docking scores, its lower effectiveness compared to Galantamine suggests that further optimization is required for enhanced efficacy. These findings underscore the potential of peanut oil in pharmaceutical development, with compounds like oleic acid and oxiraneoctanoic acid emerging as promising candidates for continued research and drug development. Peanut oil from Algeria holds significant promise for future applications in antioxidant, neuroprotective, and anticancer therapies.

## Graphical Abstract

**Figure:**
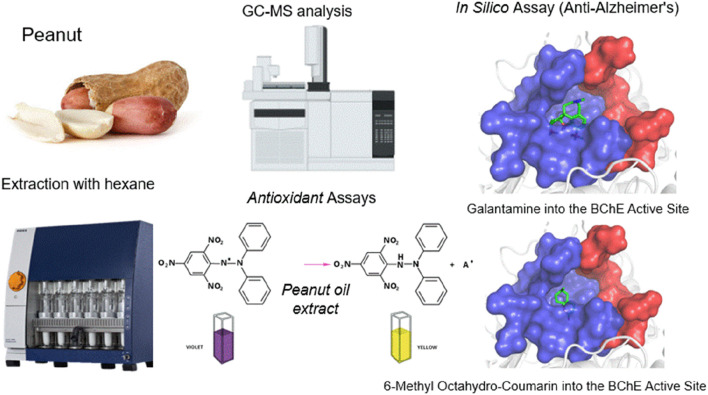

## 1 Highlights

• Algerian peanut oil contains 20 compounds; oleic acid is the most abundant at 41.98%.
• Peanut oil demonstrates antioxidant effects with an IC_50_ value of 265.96 μg/mL.
• Peanut oil inhibits butyrylcholinesterase, with 6-methyl Octahydro-Coumarin.
• Oleic and palmitic acids in peanut oil target melanoma and carcinoma cell lines.
• Oxiraneoctanoic acid in peanut oil shows potential against leukemia cell lines.

## 2 Introduction

Medicinal and edible plants have long been integral to both traditional and modern medicine, offering a rich source of bioactive compounds with therapeutic potential. These plants have historically been used to treat a variety of ailments, and their relevance continues to grow in the context of drug discovery and functional food development. Bioactive components, including phenolics, flavonoids, terpenes, and essential oils, are recognized for their ability to combat oxidative stress, inflammation, and metabolic disorders, promoting overall health (Kavi Kishor et al., 2020; Singh et al., 2021). As the prevalence of chronic conditions like cardiovascular diseases, diabetes, and neurodegenerative disorders increases, the role of medicinal plants in pharmaceutical and nutritional sciences becomes even more crucial (Chen et al., 2023). The continued exploration of their bioactive compound’s aids in the prevention and management of these conditions, offering a natural approach to health maintenance. Among these plants, peanuts (*Arachis hypogaea*) are particularly notable for their rich nutritional content and potential health benefits, making them a focus of both nutritional and medicinal research (Kavi Kishor et al., 2020; Singh et al., 2021).

Peanuts (*A. hypogaea*), in particular, stand out not only for their nutritional value but also for their medicinal potential. Rich in bioactive compounds, peanuts provide a significant source of essential nutrients and healthy fats. Comprising 40%–50% fatty oil and 29.59% protein, they offer high-quality plant-based protein and contribute to cardiovascular health through their monounsaturated fat content, which has been linked to lowering LDL cholesterol levels (Chen et al., 2023; Cui et al., 2023). Additionally, peanuts contain potent antioxidants, including resveratrol and phenolic acids, which help combat oxidative stress and inflammation, potentially reducing the risk of chronic diseases (Kavi Kishor et al., 2020; Cui et al., 2023). These bioactive compounds make peanuts a valuable addition to functional foods and dietary supplements, as they may play a role in preventing metabolic disorders, such as diabetes, and supporting overall wellbeing (Kavi Kishor et al., 2020; Vikal et al., 2024).

While peanuts have been extensively studied for their nutritional benefits, research on their specific therapeutic properties in relation to certain health conditions remains limited. Emerging studies suggest that peanuts and their derivatives may offer benefits for metabolic health, such as improved weight management and a reduced risk of type 2 diabetes (Vikal et al., 2024). Furthermore, certain compounds found in peanuts, such as fatty acids and phenolics, show potential in neuroprotective research, particularly in relation to brain health and the prevention of neurodegenerative diseases like Alzheimer’s disease (Franco et al., 2023). However, there is a gap in the literature regarding the antioxidant and anti-Alzheimer’s properties of peanut oil. To address this, gas chromatography-mass spectrometry (GC-MS) can be employed to identify the chemical components of peanut oil and assess its therapeutic properties, as GC-MS is a powerful tool for analyzing plant oils and identifying bioactive compounds, including fatty acids, terpenes, and phenolics (Shoaib et al., 2023).

This study represents the first comprehensive investigation into the antioxidant and anti-Alzheimer’s potential of peanut oil extracted from Algerian peanuts (*A. hypogaea* L.), filling a critical gap in the literature. By combining *in vitro* and *in silico* methodologies, this research not only evaluates the biological activity of the oil but also provides mechanistic insights into its potential health benefits. The *in vitro* analysis involves standard antioxidant assays, such as DPPH (2,2-diphenyl-1-picrylhydrazyl) and CUPRAC (cupric ion reducing antioxidant capacity), to measure the oil’s ability to neutralize free radicals (Rahim et al., 2023; Munteanu and Apetrei, 2021). Additionally, its potential in mitigating neurodegenerative processes is evaluated through enzyme inhibition assays, particularly focusing on butyrylcholinesterase (BChE), a key enzyme involved in Alzheimer’s disease (Tuzimski and Petruczynik, 2022; Hajlaoui et al., 2021; Mutlu et al., 2023; Cancela et al., 2020; Indrayanto et al., 2021; Agamah et al., 2020). Furthermore, molecular docking simulations offer insights into the binding interactions between peanut oil compounds and BChE, complementing the *in vitro* findings. This study provides the first detailed report on the bioactive properties of Algerian peanut oil, highlighting its potential as a source of neuroprotective and antioxidant compounds.

## 3 Experimental

### 3.1 Sample preparation and oil extraction

Peanut seeds were meticulously hand-harvested from the El Oued region in Algeria. The seeds were dried at room temperature for 1 month and then conserved at 4°C until their use. About 1 g of dried seeds was ground into a coarse powder to optimize the surface area for efficient extraction. This powder was then subjected to Soxhlet extraction (FOSS Soxtec™ 8,000) using n-hexane (≥98% purity, Sigma-Aldrich) as the solvent, chosen for its efficacy in oil extraction. The extraction process spanned 6 h, ensuring thorough oil recovery. For each 1 g of peanut kernels placed in the thimbles, 25 mL of n-hexane was poured into the extraction vessels. To maintain the oil’s integrity and prevent degradation, it was stored in amber glass bottles at a controlled temperature of 4°C, minimizing light exposure until further analysis.

### 3.2 Gas chromatography-mass spectrometry (GC-MS) analysis

The lipophilic extract obtained from peanut underwent a methylation process prior to GC-MS analysis, as the fatty acids in their original form are not sufficiently volatile for direct gas chromatographic injection. The methylation step converts the fatty acids into fatty acid methyl esters (FAMEs), which are more suitable for gas chromatography due to their increased volatility. Briefly, the extracted peanut oil (1 mL) was subjected to a methylation process to convert the fatty acids into their corresponding fatty acid methyl esters (FAME). Since the oil primarily contains triacylglycerols, which are not directly analyzable by GC-MS, the lipids were first reacted with 2 mL of methanol and 200 µL of concentrated sulfuric acid (98%) as a catalyst. This mixture was heated at 70°C for 2 h to complete the esterification reaction. After cooling, the FAME were extracted by adding 2 mL of hexane and 1 mL of distilled water. The organic phase (hexane) containing the FAME was separated and washed with a saturated sodium chloride solution (1 mL) to remove any remaining water-soluble impurities. The FAME were then dried over anhydrous sodium sulfate (0.2 g) and filtered before being concentrated to 1 mL under a stream of nitrogen gas.

GC-MS analysis was performed with a Hewlett-Packard 6,890 gas chromatograph coupled with a Hewlett-Packard 5,973 mass spectrometer. The GC system featured a capillary column (60 m × 0.32 mm inner diameter, 0.25 µm film thickness) and a flame ionization detector (FID). Helium served as the carrier gas at a flow rate of 1 mL/min. The temperature program started at 60°C, increased to 250°C at a rate of 3°C/min, and was held at 250°C for 10 min. The detector and injector temperatures were set at 245°C and 250°C, respectively. The mass spectrometer was operated in electron ionization mode with an ionization potential of 70 eV. Compound identification was achieved by comparing retention times and mass spectra with NIST and Wiley libraries, and matching fragmentation patterns and retention indices which were reported in the literature.

### 3.3 Phytochemical profiling of the extracted peanut oil

#### 3.3.1 Total phenolic content (TPC)

TPC was determined using the Folin-Ciocalteu method (Singleton and Rossi, 1965). In a 96-well microplate, 20 µL of peanut oil was mixed with 100 µL of Folin-Ciocalteu reagent (Sigma-Aldrich) and 75 µL of sodium carbonate solution (7.5%, ACS reagent, ≥99.5%, Sigma-Aldrich). The mixture was incubated in the dark for 30 min, and absorbance was measured at 765 nm using a microplate reader (Perkin Elmer EnSpire, 2,300 Multilabel Multimode Microplate Reader). The phenolic content in the peanut oil was expressed as micrograms of gallic acid equivalent (GAE) per milligram of oil, providing a measure of the antioxidant potential of the oil based on its phenolic concentration.

#### 3.3.2 Total flavonoid content (TFC)

TFC was measured following (Topçu et al., 2007). To 50 µL of peanut oil, 130 µL of methanol (≥99.8%, ACS reagent, Sigma-Aldrich), 10 µL of potassium acetate (Reagent Plus^®^, ≥99.0%, Sigma-Aldrich), and 10 µL of aluminium chloride (Reagent Plus^®^, 99%, Sigma-Aldrich) were added. After a 30-min incubation at room temperature, absorbance was read at 415 nm using a microplate reader. Quercetin (≥95% (HPLC), Sigma-Aldrich) was used as the standard for constructing a calibration curve, and it was treated identically to the oil samples. The TFC was expressed as milligrams of quercetin equivalent (QE) per Gram of oil, providing a measure of the concentration of flavonoids, which are known for their antioxidant and anti-inflammatory properties.

### 3.4 Antioxidant activity

#### 3.4.1 DPPH radical scavenging activity

The DPPH radical scavenging activity test is important for assessing the antioxidant potential of a substance. It measures the ability of compounds to neutralize free radicals, specifically the DPPH radical, which is stable and produces a purple color. The DPPH radical scavenging activity was assessed following (Tel et al., 2012), with modifications. In a 96-well microplate, 160 µL of methanolic DPPH (Sigma-Aldrich) solution was mixed with 40 µL of peanut oil at various concentrations. Ethanol (≥99.9% (GC), Sigma-Aldrich) was used as a negative control, replacing the peanut oil, while known antioxidants, butylated hydroxytoluene (BHT) and butylated hydroxyanisole (BHA), both from Sigma-Aldrich, served as positive controls. These standards were processed in the same manner as the samples. Following a 30-min incubation period in the dark to allow for reaction completion, absorbance was measured at 517 nm using a microplate reader. The inhibition of DPPH radicals by the peanut oil and standards was calculated using the following formula:

$$\text{Inhibition }\left(\%\right)=\left[\left({\text{Abs}}_{\text{Blank}}\;–\;{\text{Abs}}_{\text{Sample}}\right)/{\text{Abs}}_{\text{Blank}}\right]*100$$

Abs_Blank_: absorbance of control reaction; Abs_Sample_: absorbance of the test sample. Tests were carried out in triplicates.

#### 3.4.2 Cuprac reducing antioxidant capacity (CUPRAC)

The CUPRAC assay is used to assess the antioxidant capacity of a substance by measuring its ability to reduce copper (II) ions (Cu^2^⁺) to copper(I) ions (Cu⁺). In this test, antioxidants in the sample react with copper (II) ions, reducing them to copper(I), which forms a colored complex that can be detected spectrophotometrically. The intensity of the color change correlates with the antioxidant capacity of the sample (Apak et al., 2008). In a 96-well microplate, 60 µL of ammonium acetate buffer (7.7%, ACS reagent, ≥97%, Sigma-Aldrich), 50 µL of neocuproine (≥98%, Sigma-Aldrich) (0.156%), and 50 µL of cupric chloride solution (0.170%, ACS reagent, ≥99.0%, Sigma-Aldrich) were mixed with 40 µL of peanut oil at different concentrations. The positive controls, BHT (butylated hydroxytoluene) and BHA (butylated hydroxyanisole), both from Sigma-Aldrich, were treated in the same manner as the peanut oil extracts. Following a 1-h incubation period at room temperature, the absorbance of the resulting-colored complex was measured at 450 nm using a microplate reader. The absorbance readings were used to calculate the reducing power of the peanut oil and compared with the standards to assess its antioxidant capacity.

### 3.5 Anti-alzheimer’s activity *via* BChE inhibition assay

The inhibition of Butyrylcholinesterase (BChE) is vital for assessing the potential of peanut oil constituents to impede the breakdown of acetylcholine, a neurotransmitter closely associated with the progression of Alzheimer’s disease. BChE inhibition was evaluated using (Ellman et al., 1961). In a 96-well microplate, 20 µL of BChE from equine serum (≥10 units/mg protein, E.C. 3.1.1.8, Sigma-Aldrich) solution (6.85 × 10^−3 ^U) was mixed with 150 µL of sodium phosphate buffer (pH 8.0) and 10 µL of peanut oil at various concentrations. After a 15-min incubation at 25°C, 10 µL of DTNB [5,5′-dithio-bis(2-nitrobenzoic) acid] (≥98%, BioReagent, Sigma-Aldrich) (0.5 mM) and 10 µL of butyrylcholine iodide (≥98%, Sigma-Aldrich) (0.2 mM) were added. Absorbance was measured at 412 nm initially and again after 15 min using a microplate reader. Galantamine, a known BChE inhibitor, was used as a positive control and treated in the same manner as the peanut oil extracts. The percentage of BChE enzyme inhibition was calculated using the following formula:

$$\text{Inhibition }\left(\%\right)=\left[\left({\text{Abs}}_{\text{Control}}\;–\;{\text{Abs}}_{\text{Sample}}\right)/{\text{Abs}}_{\text{Control}}\right]*100$$

$${\text{Abs}}_{\text{Control}}:\text{BChE enzyme}-\text{extract};\;{\text{Abs}}_{\text{Sample}}:\text{BChE enzyme}+\text{oil}$$

### 3.6 Statistical analysis

Statistical analyses were conducted to evaluate the significance of the experimental results. Linear regression analysis was applied to determine the half-maximal inhibitory concentration (IC₅₀) and A₀.₅ values, which represent the concentration of peanut oil or reference compounds required to achieve 50% inhibition or antioxidant activity, respectively. To identify significant differences between groups, one-way analysis of variance (ANOVA) was performed, with a significance threshold set at *p* < 0.05. When significant differences were detected, the Tukey *post hoc* test was used for multiple comparisons. All statistical analyses were performed using XLSTAT software. Results are presented as the mean ± standard deviation (SD) of three independent measurements.

### 3.7 Physicochemical, ADMET, cytotoxic, and anticancer properties determination

Canonical SMILES for the identified compounds, including Oleic acid, Palmitic acid, Stearic acid, and Oxiraneoctanoic acid, 3-octyl, were retrieved from the PubChem database (NCBI, 2023). Physicochemical properties were predicted using Lipinski’s rule of five on the Swiss ADME platform (SwissADME, 2023), assessing parameters such as size, polarity, lipophilicity, solubility, flexibility, and unsaturation. ADMET properties were analyzed using the admetSAR 2.0 platform (IMMD, 2023), focusing on human intestinal absorption (HIA), Caco-2 permeability, blood-brain barrier (BBB) permeability, and human bioavailability (HOB). Cytotoxic and anticancer effects of the compounds on tumor cells were evaluated using the CLC-Pred server (Way2drug, 2023). The compounds extracted from peanut oil were evaluated for their anticancer activity against a variety of cancer cell lines, including melanoma A2058, metastatic melanoma SK-MEL-1, non-small cell lung cancer NCI-H838, lung carcinoma DMS-114, ovarian adenocarcinoma IGROV, and leukemia H9. These assays aimed to assess the cytotoxic potential of the oil’s major constituents, with each compound demonstrating varying degrees of effectiveness across the different cancer type. This prediction evaluates the probability of activity (Pa) *versus* inactivity (Pi), with a higher Pa score indicating a greater likelihood of activity compared to inactivity, though not guaranteeing it. This combined approach aims to identify promising compounds for further experimental validation, facilitating the evaluation of their therapeutic potential in cancer treatment.

### 3.8 Molecular docking for Anti-Alzheimer’s activity

Molecular docking analysis was performed on key compounds identified in peanut oil to evaluate their binding affinities with Butyrylcholinesterase (BChE), aiming to assess their potential anti-Alzheimer’s activity. The 3D structure of BChE (PDB ID: 2XQF) was obtained from the Protein Data Bank and processed for docking using LeadIT 2.1.8 (Biosolveit, 2023). All heteroatoms, cofactors, and non-essential water molecules were removed from the crystallized structure, and any missing atoms were added. The active site was defined by residues within a 6.5 Å radius around the inhibitor, and additional residues critical for maintaining the binding cavity were included. The protonation states and side-chain orientations were optimized. Ligand structures were prepared using LigPrep in Schrödinger’s Maestro 11.3 (Schrodinger Release, 2015-1, 2015), considering all possible tautomers and protonation states. Molecular docking was carried out using FlexX 2.1.8 (Rarey et al., 1996), with binding energies (ΔG in kJ/mol) indicating the strength of interactions. The co-crystallized ligand was redocked to validate docking parameters, ensuring an RMSD value of less than 2 Å.

## 4 Results and discussion

### 4.1 Yield of extraction, total phenolic, and flavonoid contents

Plants have long been central to medicinal research, with peanut oil providing valuable insights into its composition and health benefits. Despite its traditional use, studies on peanut oil remain relatively limited (Djeghim et al., 2021; Djeghim et al., 2022). This study highlights that the hexane extraction method is particularly effective for obtaining higher yields of bioactive compounds. Hexane excels at extracting non-polar substances like lipids and specific phytochemicals due to its low viscosity and high volatility (Bhuiya et al., 2020). Improved yields with hexane extraction depend on factors such as solvent-to-solute ratio and extraction techniques, allowing for the capture of a broader range of bioactive compounds essential for unlocking potential health benefits (Shad et al., 2012; Zio et al., 2021). The oil extracted from peanuts exhibited a high yield of 51.85% ± 1.76%, indicating an efficient process that produces a substantial amount of oil from the seed material. The extracted oil was noted for its uncolored appearance and pleasant odor, attributed to the presence of methyl nonyl ketone. Analysis of the oil revealed noteworthy levels of bioactive compounds, with a total phenolic content measured at 28.21 ± 2.43 µg GAE/mg, indicating a rich presence of phenolic constituents known for their antioxidant properties. Similarly, the total flavonoid content was found to be 27.72 ± 1.25 µg QE/mg, underscoring the oil’s potential health benefits given the antioxidant and anti-inflammatory effects of flavonoids (Table 1).

**TABLE 1:** The yield of extraction, total phenolic content, and total flavonoid content in oil extracted from peanuts.

Sample	Phenolic content (µg GAE/mg)	Flavonoid content (µg QE/mg)	Fat content (%)
Peanut Oil	28.21 ± 2.43	27.72 ± 1.25	51.85 ± 1.76

µg QE/mg: µg quercetin equivalent per mg; µg GAE/mg: µg gallic acid equivalent per mg.

### 4.2 Phytochemcial profiling of the peanuts oil

**FIGURE 1:**
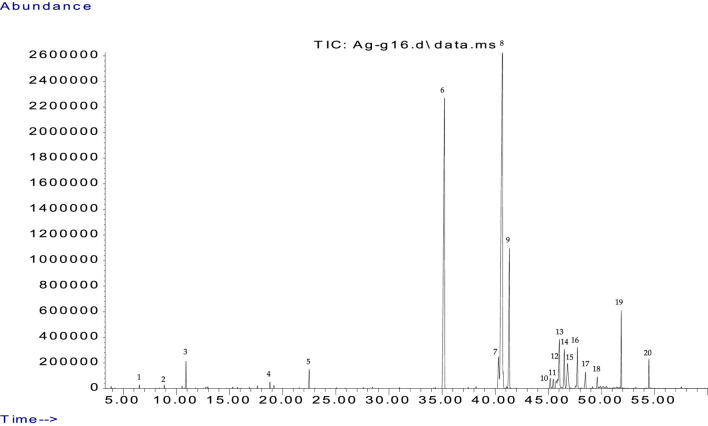
GC-MS chemical profile of the oil extracted from peanut (*Arachis hypogaea* L.).

GC-MS analysis of peanut oil provided a comprehensive fatty acid profile, accounting for 99.9% of its composition (Figure 1). The predominant fatty acid identified was oleic acid (41.98%), followed by palmitic acid (26.90%) and stearic acid (8.11%), which is consistent with prior research (Nile and Park, 2013). This detailed breakdown illustrates the balance of various fatty acids, categorized into saturated fatty acids (SFA), monounsaturated fatty acids (MUFA), and polyunsaturated fatty acids (PUFA), each contributing to the oil’s nutritional and functional properties (Table 2). To ensure the accuracy and reliability of GC-MS analysis, the retention indices of the identified compounds were recalculated using the Van Den Dool-Kratz equation (Zhokhov et al., 2018). This involved applying the retention times of standard n-alkanes to derive the indices for each compound. Subsequently, these recalculated indices were compared with those reported in the NIST and Adams literature databases.

(1) Major Compounds: The main fatty acid in peanut oil is oleic acid, which makes up 41.98% of its total fatty acid content. This monounsaturated fatty acid is recognized for its heart health benefits, such as lowering LDL cholesterol and raising HDL cholesterol. This makes it important for promoting cardiovascular health, reducing inflammation, and helping to maintain cell membrane fluidity and improve insulin sensitivity (Lopez-Huertas, 2010; Yin et al., 2007; Zhao et al., 2019; Anavi-Cohen et al., 2023). The second most common fatty acid is palmitic acid, which comprises 26.90% of the oil. While palmitic acid is a saturated fatty acid linked to raising LDL cholesterol levels, its effects can be lessened by the presence of oleic acid and other beneficial compounds in peanut oil (Astrup et al., 2011; Bhuiya et al., 2020; Shad et al., 2012). Stearic acid accounts for 8.11% of the oil and is another important saturated fatty acid. Unlike palmitic acid, stearic acid does not negatively affect LDL cholesterol levels and may even help lower them, thus supporting heart health (Tong et al., 2020; Slavin, 2004). Stearic acid’s neutral effect on cholesterol makes it a promising area for further research into its benefits for lipid profiles.
(2) Minor Compounds: The remaining composition includes a variety of minor fatty acids and compounds. For example, n-Caprylic acid and n-Capric acid, though present in smaller amounts, contribute to the oil’s flavor and aroma (Arai et al., 1966). Oxiraneoctanoic acid and Azela aldehydic acid, detected in trace amounts, may impart additional health benefits such as antioxidant and antimicrobial properties, enhancing the oil’s overall functional attributes (Kenar et al., 2017; Al-Snafi, 2020).
(3) Traces and Additional Insights: The trace compounds, including 4-Methyl-exo-tricyclo [6.2.1.0 (2.7)] undecane and 6-Methyl Octahydro Coumarin, might play a role in stabilizing the oil and potentially influencing its sensory properties. Compounds such as Iso-Iridomyrmecin and Carbamic acid could further contribute to the oil’s complex chemical profile, affecting its biological activity and stability (Lončar et al., 2020; Stökl et al., 2012; Garofalo et al., 2011).
(4) Nutritional and Functional Implications: The oil’s profile shows a well-balanced distribution of fatty acids, with 47.18% SFA, 46.94% MUFA, and 2.38% PUFA. The high proportion of MUFAs supports the oil’s role in reducing cardiovascular risk and improving lipid profiles. The relatively low PUFA content is beneficial as it minimizes the risk of oxidation, making the oil stable for cooking and longer shelf life.

**TABLE 2:** Chemical composition of the oil extracted from peanut (*Arachis hypogaea* L.).

PK	Compound	Area (%)	Retention index	Index (NIST/Adams)	Comparison
1	n-Caproic acid	0.1103	658	656	Match
2	Heptanoic acid	0.0963	882	880	Match
3	n-Caprylic acid	0.6534	1,089	1,090	Match
4	Azela aldehydic acid	0.2702	1880	1875	Match
5	Nonanedioic acid	0.8979	2,245	2,240	Match
6	Palmitic acid	26.9063	3,520	3,515	Match
7	α-Linolenic acid	2.3780	4,025	4,020	Match
8	Oleic acid	41.9856	4,070	4,065	Match
9	Stearic acid	8.1192	4,135	4,130	Match
10	4-Methyl-exo-tricyclo [6.2.1.0 (2.7)] undecane	0.5910	4,510	4,515	Match
11	6-Methyl Octahydro Coumarin	1.0992	4,550	4,540	Match
12	Iso-Iridomyrmecin	0.7052	4,570	4,560	Match
13	Oxiraneoctanoic acid, 3-octyl	3.4956	4,600	4,605	Match
14	Oxiraneoctanoic acid	2.6535	4,650	4,640	Match
15	E, E, Z-1,3,12-Nonadecatriene-5,14-diol	2.6759	4,680	4,675	Match
16	Eicosanoic acid	2.3079	4,770	4,775	Match
17	(2R)-2-(1,3-Dithian-2-yl) isoborneol	1.0815	4,850	4,845	Match
18	Carbamic acid	0.5976	4,950	4,940	Match
19	Docosanoic acid	2.4349	5,185	5,180	Match
20	Tetracosanoic acid	0.9407	5,445	5,440	Match
Summary					
✓ Saturated Fatty Acids (SFA): 47.18%					
✓ Unsaturated Fatty Acids (UFA): 49.31%					
✓ Monounsaturated Fatty Acids (MUFA): 46.94%					
✓ Polyunsaturated Fatty Acids (PUFA): 2.38%					
✓ Others: 3.90%					
✓ Total: 99.90%					

### 4.3 *In Vitro* antioxidant activity

The antioxidant potential of the extracted peanut oil was assessed using the DPPH radical scavenging assay and the CUPRAC assay. As shown in Table 3, peanut oil exhibited relatively weak antioxidant activity in both tests, with an IC_50_ value of 1715.92 ± 93.28 μg/mL for the DPPH assay and an A_0.5_ value of 265.96 μg/mL for the CUPRAC assay. These values are significantly higher (*p < 0.001*) than those of the reference compounds BHA (IC_50_: 15.68 ± 0.25 μg/mL; A_0.5_: 1.79 ± 0.07 μg/mL) and BHT (IC_50_: 6.68 ± 0.06 μg/mL; A_0.5_: 4.58 ± 0.10 μg/mL), indicating a much lower antioxidant potential for peanut oil. This indicates that, despite the presence of phenolic and flavonoid compounds, their concentrations or types may not effectively neutralize free radicals or reduce metal ions. Additionally, peanut oil has lower antioxidant activity compared to oils like sesame and olive oil (Prabasheela et al., 2015). The higher IC_50_ value in the DPPH assay suggests reduced radical scavenging ability, likely due to the loss of hydrophilic antioxidants during extraction. While peanut oil contains polyphenolic compounds, their effectiveness is diminished by non-polar lipids, unlike oils rich in polyunsaturated fatty acids, which typically show higher antioxidant activity due to compounds like tocopherols (Kapoor et al., 2021; Phuah et al., 2022). Moreover, interactions between tocopherols and phenolic acids may further lessen antioxidant activity (Palafox-Carlos et al., 2012).

**TABLE 3:** The antioxidant ability of oil extracted from peanuts.

Samples	DPPH	CUPRAC
	IC_50_ (µg/mL)	A_0.5_ (µg/mL)
Peanut Oil	1715.92 ± 93.28^***^	265.96 ± 14.85^***^
BHA^a^	15.68 ± 0.25^*^	1.79 ± 0.07^*^
BHT^a^	6.68 ± 0.06^*^	4.58 ± 0.10^*^

^a^ Reference compound.

***p < 0.001.

### 4.4 *In Vitro* Alzheimer’s activity

Anti-Alzheimer’s activity is often linked to the inhibition of Butyrylcholinesterase (BChE), an enzyme responsible for breaking down acetylcholine, a neurotransmitter crucial for memory and cognitive function. In Alzheimer’s disease, reduced acetylcholine levels contribute to cognitive decline, making BChE inhibition a potential therapeutic strategy to help maintain acetylcholine levels in the brain. The inhibitory effect of the peanut oil on BChE was measured and compared to the standard inhibitor, Galantamine. The data, presented in Table 4, show that the peanut oil demonstrated a weak BChE inhibitory capacity. At a concentration of 200 μg/mL, the oil achieved an inhibition percentage of 36.47% ± 3.71% which was significantly lower compared to Galantamine 96.98 ± 0.94 μg/mL, as indicated by ** (*p = 0.00028*), at the same concentration and had an IC_50_ value of 11.61 ± 0.22 μg/mL. The observed inhibition by peanut oil, although limited, is still relevant to its potential anti-Alzheimer’s activity, as even partial inhibition of BChE could help sustain acetylcholine levels. To date, there have been no reports in the literature on the BChE inhibitory capacity of Algerian peanut oil, making this a novel finding. These results are in line with previous studies, such as one by (Bozorgi et al., 2021), which showed that the aqueous extract of areca nuts exhibited strong anti-Alzheimer capacity through inhibition of amyloid β aggregation. Similarly, (Chauhan and Chauhan, 2020), highlighted the protective effects of walnut-enriched diets against AD.

**TABLE 4:** Inhibition of butyrylcholinesterase (BChE) by peanut oil.

Sample	BChE inhibition (%) at 200 μg/mL	IC_50_ (µg/mL)
Peanut Oil	36.47 ± 3.71 **	>200
Galantamine^a^	96.98 ± 0.94 *	11.61 ± 0.22

Values expressed are means ± S. D, of three parallel measurements.

**p < 0.001.

^a^ Reference compound.

### 4.5 Physicochemical, cytotoxic and anticancer properties of peanut oil compounds

The physicochemical and ADMET (Absorption, Distribution, Metabolism, Excretion, and Toxicity) profiles of compounds found in peanut oil, along with their cytotoxic effects, are critical for evaluating the therapeutic potential of these compounds, particularly in targeting various cancer cell lines. The analysis combines cytotoxicity data with the physicochemical characteristics of the compounds, offering a comprehensive overview of their anticancer activities.

Table 5 summarizes the properties of the molecules identified in peanut oil, detailing their molecular weight, chemical formula, structure, and canonical SMILES. All compounds comply with Lipinski’s Rule of Five, suggesting good oral bioavailability. Notably, oxiraneoctanoic acid, 3-octyl, with a log *p*-value below 5, demonstrates favorable lipophilicity, highlighting its potential as a drug-like candidate. This thorough analysis emphasizes the fundamental characteristics of these compounds, with a special focus on oxiraneoctanoic acid, 3-octyl, for its promising prospects in drug development.

**TABLE 5:** Molecules identified in peanut oil: Molecular formula, structure, and canonical SMILES.

N°	Molecule	Molecular formula	Structure
1	Oleic acid	C_18_H_34_O_2_	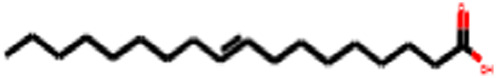
2	Palmitic acid	C_16_H_32_O_2_	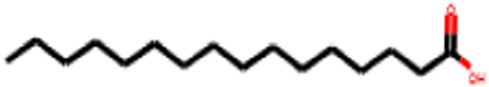
3	Stearic acid	C_18_H_36_O_2_	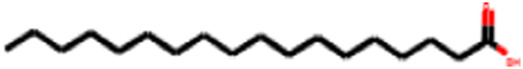
4	Oxiraneoctanoic acid, 3-octyl	C_18_H_34_O_3_	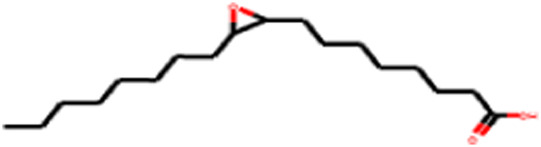

Table 6 presents the ADMET profiles of the major compounds in peanut oil, offering insights into their pharmacokinetic and toxicological characteristics. Each compound exhibited excellent absorption capabilities, effectively crossing the human intestinal barrier (HIA+), Caco-2 cell layer, and blood-brain barrier (BBB). All four compounds showed positive HIA+ and Caco-2 permeability, indicating strong potential for effective oral administration and systemic bioavailability. Importantly, none of the compounds acted as substrates or inhibitors of P-glycoproteins, which play a significant role in drug resistance.

**TABLE 6:** Pharmacokinetic properties of molecules determined in peanut oil using Swiss ADMETSAR 2.0 server.

Parameters	Oleic acid	Palmitic acid	Stearic acid	Oxiraneoctanoic acid, 3-octyl
Absorption				
HIA	HIA+	HIA+	HIA+	HIA+
Caco-2	+	+	+	+
BBB	+	+	+	+
HOB	–	–	–	–
P-glycoprotein substrate	–	–	–	–
P-glycoprotein inhibitor	–	–	–	–
Distribution				

Subcellular Localization	Plasma membrane	Mitochondria	Mitochondria	Mitochondria
Metabolism				
CYP3A4 substrate	–	–	–	–
CYP2C9 substrate	+	+	+	+
CYP2D6 substrate	–	–	–	–
CYP3A4 inhibition	–	–	–	–
CYP2C9 inhibition	–	–	–	–
CYP2C19 inhibition	–	–	–	–
CYP2D6 inhibition	–	–	–	–
CYP1A2 inhibition	+	+	+	–
CYP inhibitory promiscuity	–	–	–	–
UGT catalyzed	–	–	–	–
Elimination				
OCT2 inhibitor	–	–	–	–
MATE1 inhibitor	–	–	–	–
OATP1B3 inhibitor	–	–	–	+
OATP1B1 inhibitor	–	+	+	+
BSEP inhibition	–	–	–	–
OATP2B1 inhibitor	–	–	–	–
Toxicity				
Eye corrosion	+	+	+	–
Eye irritation	+	+	+	+
Carcinogenicity	–	–	–	–
Ames mutagenesis	–	–	–	–
Hepatotoxicity	–	+	+	+
Skin sensitization	+	+	+	–
Respiratory toxicity	–	–	–	–
Mitochondrial toxicity	+	+	+	–
Nephrotoxicity	–	–	–	+
AOT (c)	Class IV	Class IV	Class IV	Class III
Estrogen receptor binding	–	–	–	–
Thyroid receptor binding	–	+	–	+
ADMET Properties-Regression				
Water Solubility (log S)	−4.04	−3.502	−3.502	−3.384
PPB (100%)	0.518	0.433	0.433	0.67
AOT (log (1/(mol/kg)))	1.246	1.16	1.125	1.7
Tetrahymenapyriformis (pIGC50 (µg/L))	1.324	1.45	1.585	1.394

HIA: human intestinal absorption; BBB: blood brain barrier; HOB: human oral bioavailability; PPB: plasma protein binding; AOT: acute oral toxicity; UGT, catalyzed: UDP-glucuronosyl transferase.

The ADMET analysis indicates that oleic acid localizes in the plasma membrane, while palmitic acid, stearic acid, and oxiraneoctanoic acid, 3-octyl, localize in the mitochondria, which may influence their biological effects. Most compounds inhibited CYP1A2, except for oxiraneoctanoic acid, 3-octyl, indicating significant hepatic metabolism for the majority. None inhibited CYP3A4 or CYP2D6, which reduces the risk of drug-drug interactions. Toxicity studies revealed that oleic, palmitic, and stearic acids can cause eye corrosion, skin irritation, and mitochondrial toxicity, while oxiraneoctanoic acid, 3-octyl, showed no such effects. Hepatotoxicity was noted for palmitic, stearic, and oxiraneoctanoic acids, while nephrotoxicity was associated with oxiraneoctanoic acid, 3-octyl. Plasma protein binding was moderate for all compounds, with oxiraneoctanoic acid, 3-octyl, demonstrating the highest binding affinity. Oleic, palmitic, and stearic acids were classified as low toxicity (Class IV), while oxiraneoctanoic acid, 3-octyl, was classified as moderate toxicity (Class III).

Extracting bioactive compounds or fractions is a critical first step in traditional medicinal plant research, followed by qualitative and quantitative analyses to identify the active components (Ceylan et al., 2015). However, this process is often time-consuming and costly (Bucao and Solidum, 2021). To improve research efficiency, modifications to traditional approaches have been explored. As computer technologies advance, *in silico* methods such as the simulation of compound-target interactions have become increasingly reliable and accurate (Ntie-Kang et al., 2013). These computational approaches allow for the rapid identification of potential relationships between compounds and biological targets, facilitating faster drug discovery. One such advancement is network pharmacology, a technology that enables researchers to explore complex interactions between multiple compounds and their diverse activity targets. For this reason, the objective of this study was shaped by the promise of network pharmacology, aiming to apply these modern tools to streamline the discovery process and understand the multifaceted interactions of bioactive compounds in peanut oil.

The cytotoxicity data in Table 7 highlight the strong antitumor potential of the primary fatty acids in peanut oil, including oleic acid, palmitic acid, stearic acid, and oxiraneoctanoic acid, 3-octyl. Oleic acid exhibited significant cytotoxic effects across various cancer cell lines, especially against melanoma (A2058 and SK-MEL-1) and non-small cell lung cancer (NCI-H838), indicating broad-spectrum antitumor activity. Palmitic acid also showed notable cytotoxicity, particularly against lung carcinoma and metastatic melanoma. Stearic acid mirrored the cytotoxic profile of palmitic acid, reinforcing its potential against melanoma and lung cancers.

**TABLE 7:** Cytotoxic effect of the selected compounds against tumor cells using the online server CLC-Pred.

Molecule	Pa	Pi	Cell-line (CL)	Cl full name	Tissue	Tumor type
Oleic acid	0.583	0.004	A2058	Melanoma	Skin	Melanoma
	0.563	0.011	SK-MEL-1	Metastatic melanoma	Skin	Melanoma
	0.551	0.029	NCI-H838	Non-small cell lung cancer	Lung	Carcinoma
	0.543	0.022	DMS-114	Lung carcinoma	Lung	Carcinoma
	0.508	0.018	IGROV	Ovarian adenocarcinoma	Ovary	Adenocarcinoma
Palmitic acid	0.558	0.017	DMS-114	Lung carcinoma	Lung	Carcinoma
	0.545	0.017	SK-MEL-1	Metastatic melanoma	Skin	Melanoma
	0.521	0.009	A2058	Melanoma	Skin	Melanoma
	0.537	0.033	NCI-H838	Non-small cell lung cancer	Lung	Carcinoma
Stearic acid	0.558	0.017	DMS-114	Lung carcinoma	Lung	Carcinoma
	0.545	0.017	SK-MEL-1	Metastatic melanoma	Skin	Melanoma
	0.521	0.009	A2058	Melanoma	Skin	Melanoma
	0.537	0.033	NCI-H838	Non-small cell lung cancer	Lung	Carcinoma
Oxiraneoctanoic acid, 3-octyl	0.676	0.003	H9	T-lymphoid	Hematopoietic and lymphoid	Leukemia
	0.670	0.005	DMS-114	Lung carcinoma	Lung	Carcinoma

Oxiraneoctanoic acid, 3-octyl, distinguished itself with potent activity against leukemia (H9), demonstrating specific effects on hematopoietic and lymphoid tumors, as well as significant activity against lung carcinoma. This specificity positions it as a promising candidate for targeted anticancer therapies. The combined ADMET, physicochemical, and cytotoxicity profiles suggest that these fatty acids, particularly oleic, palmitic, and stearic acids, could be valuable in treating various cancer types, while oxiraneoctanoic acid, 3-octyl, stands out for its specific potency against leukemia. This finding aligns with (Hammad et al., 2023), who reported that peanut (*A. hypogaea* L.) skin ultrasound extract exhibited significant inhibitory activity against cancer cell lines, including MCF-7, HepG-2, HCT-116, and PC-3. Additionally (Jeeunngoi et al., 2024), found that combining ethanolic peanut skin extract with either cisplatin or 5-fluorouracil had a pronounced anticancer effect on HeLa cells. Furthermore (Tsai et al., 2024), revealed that an ethyl acetate fraction of crude peanut skin extract, alongside a methanolic fraction, exhibited the highest cytotoxicity against colorectal cancer (CRC) and melanoma cells, while sparing nonmalignant human skin fibroblasts. These results support the potential of peanut oil and its constituents in cancer treatment strategies.

Table 8 outlines the findings from the docking study, assessing the physicochemical properties of key compounds from peanut oil using the Swiss ADME tool. Parameters such as molecular weight (MW), solubility (Log S), number of rotatable bonds, hydrogen bond acceptors and donors, topological polar surface area (TPSA), and consensus logP values were evaluated. Fatty acids like oleic, palmitic, and stearic acids exhibited moderate insolubility, with log S values indicating limited aqueous solubility. Their lipophilicity, reflected in consensus logP values between 5.2 and 5.93, suggests a tendency to favor lipid-rich environments, potentially influencing their bioavailability and metabolic pathways. Oxiraneoctanoic acid, 3-octyl, with a higher MW of 298.46 g/mol and a consensus logP of 4.88, presents a unique profile, characterized by a greater TPSA of 49.83 Å^2^. This suggests different interaction dynamics within biological systems compared to the more lipophilic fatty acids, indicating a distinct mechanism that could influence its activity in biological contexts.

**TABLE 8:** Determination of physicochemical properties of selected molecules using Swiss ADME.

Molecule	MW (g/mol)	Log (S)	N°-rotatable bonds	N°-H bond acceptors	N°-H bond donors	TPSA (A°)	Consensus log *p*	Physicochemical properties
Oleic acid	282.46	−5.39	15	2	1	37.30	5.71	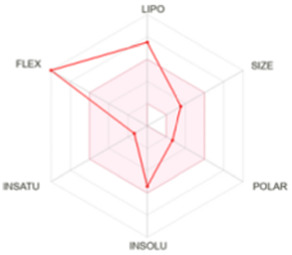
Palmitic acid	256.42	−5.31	14	2	1	37.30	5.20	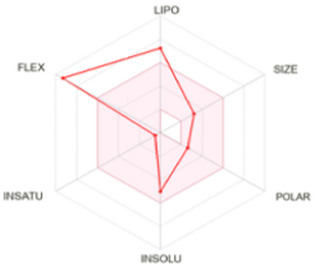
Stearic acid	284.48	−6.11	16	2	1	37.30	5.93	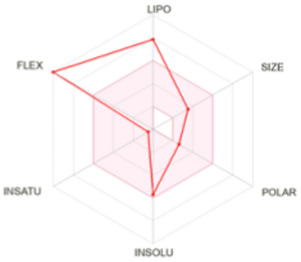
Oxiraneoctanoic acid, 3-octyl	298.46	−5.13	15	3	1	49.83	4.88	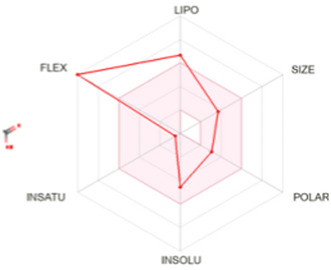

LIPO: lipophilicity, INSOLU: insolubility, INSATU: insaturation, FLEX: flexibility, POLAR: polarity.

It is important to note that the findings presented are based on *in silico* predictions and require empirical validation through laboratory experiments to confirm their accuracy. However, several studies have demonstrated the potential anticancer effects of various phytochemicals found in plant-based oils (Hammad et al., 2023; Jeeunngoi et al., 2024; Tsai et al., 2024). These bioactive compounds, such as oleic acid, palmitic acid, and stearic acid, exhibit notable cytotoxic properties against a wide range of cancer cell lines. Despite these promising results, the exact molecular pathways through which these compounds exert their anticancer effects remain underexplored. Future research involving pathway analysis and gene expression profiling will be essential to fully elucidate the mechanisms of action of these compounds and establish their true therapeutic potential.

### 4.6 Molecular Docking for Anti-Alzheimer’s activity

Molecular docking simulations of 20 primary compounds derived from peanut oil were conducted within the active site of Butyrylcholinesterase (BChE). These simulations revealed significant correlations between the experimental inhibitory activities and the docking results, as summarized in Table 9. BChE was selected for this study due to its crucial role in metabolizing acetylcholine, a neurotransmitter vital for memory and cognitive functions. In Alzheimer’s disease, acetylcholine levels are markedly reduced, contributing to cognitive decline. By inhibiting BChE, we can slow the degradation of acetylcholine, potentially aiding in maintaining its levels in the brain. Thus, targeting the BChE active site presents a promising strategy for managing neurodegenerative disorders like Alzheimer’s.

**TABLE 9:** Docking score of Galantamine and major compounds from peanuts oil within the BChE active site.

PK	Library/ID	Docking score
1	*n*-Caproic acid	−9.26
2	Heptanoic acid	−9.03
3	*n*-Caprylic acid	−9.54
4	Azelaaldehydic acid	−10.95
5	Nonanedioic acid	−11.36
6	Palmitic acid	−4.83
7	α-linolenic acid	Non
8	Oleic acid	−4.36
9	Stearic acid	Non
10	4-Methyl-exo-tricyclo [6.2.1.0 (2.7)]undecane	−6.80
11	6-Methyl OctahydroCoumarin	−15.86
12	iso-Iridomyrmecin	−14.44
13	Oxiraneoctanoic acid, 3-octyl	−9.57
14	Oxiraneoctanoic acid	−12.16
15	E,E,Z-1,3,12-Nonadecatriene-5,14-diol	−8.77
16	Eicosanoic acid	Non
17	(2R)-2-(1,3-Dithian-2-yl)isoborneol	Non
18	Carbamic acid	−10.02
19	Docosanoic acid	Non
20	Tetracosanoic acid	Non
STD	Galantamine	−23.40

STD: standard; BChE: butyrylcholinesterase; CAS: catalytic anionic site; PAS: peripheral anionic site.

**FIGURE 2:**
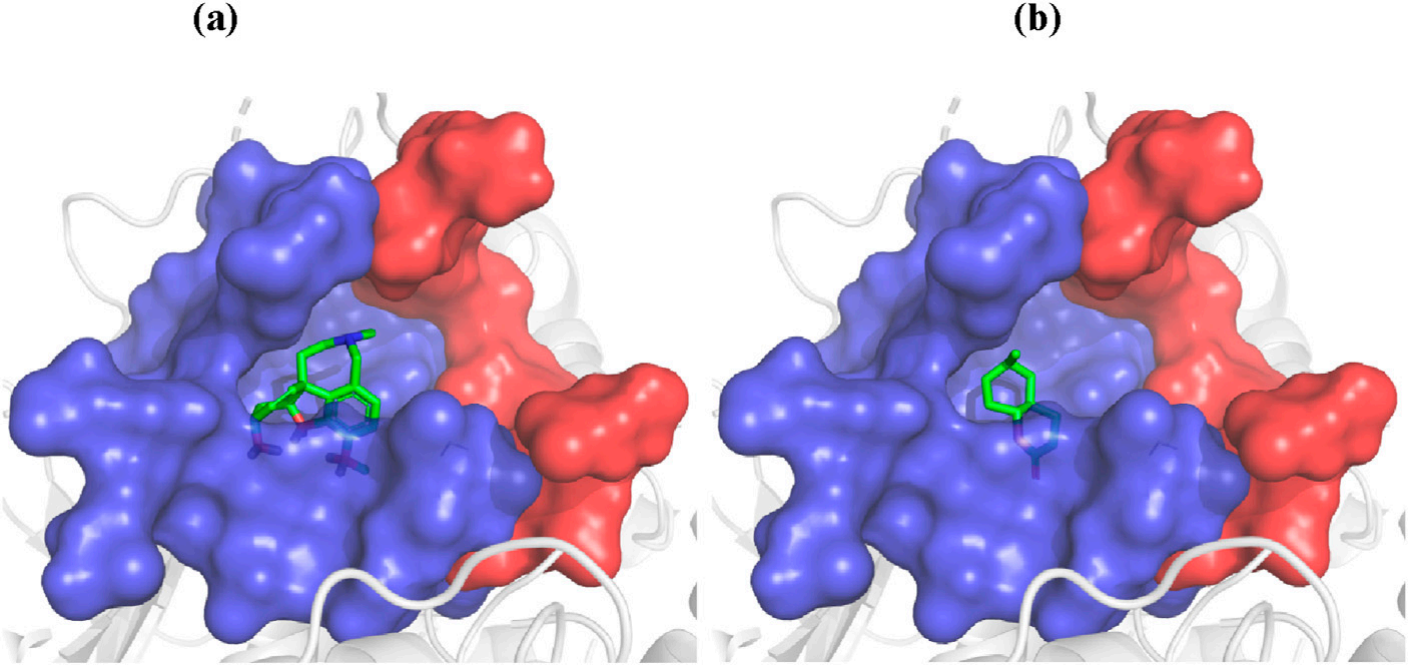
Docking of galantamine **(A)** and 6-methyl octahydro-coumarin **(B)** into the BChE active site.

The docking studies focused on two compounds: Galantamine, used as a standard, and 6-Methyl Octahydro-Coumarin derived from peanut oil, with their binding interactions illustrated in Figure 2. These simulations evaluated how each compound interacts with BChE, shedding light on their enzyme-inhibitory potential and therapeutic relevance for neurodegenerative diseases. As shown in Figure 2, both compounds predominantly bind at the bottom of the catalytic anionic site (CAS) of BChE, a region crucial for their inhibitory function. The CAS, highlighted in blue, and the peripheral anionic site (PAS), marked in red, illustrate how these compounds engage with the enzyme’s active site. The ligands, visualized with carbon atoms in green, oxygen in red, and nitrogen in blue, are strategically positioned to occupy key regions within the CAS, reinforcing their strong inhibitory potential.

Galantamine, a well-known cholinesterase inhibitor, demonstrated a docking score of −23.4 kJ/mol, indicating strong inhibitory potency consistent with its high experimental BChE inhibitory activity. In contrast, 6-Methyl Octahydro-Coumarin had a docking score of −15.86 kJ/mol, marking it as the most promising BChE inhibitor among the constituents of peanut oil. This compound was further analyzed to elucidate its binding interactions within the enzyme’s active site. Both Galantamine and 6-Methyl Octahydro-Coumarin primarily interact at the bottom of the catalytic anionic site (CAS) of BChE, a critical region for enzyme inhibition. Despite its favorable docking score, 6-Methyl Octahydro-Coumarin exhibited slightly lower inhibitory activity than Galantamine, likely due to forming fewer hydrogen bonds within the active site. Galantamine established five hydrogen bonds with key residues (Ser198, Gly116, Gly117, and His438), whereas 6-Methyl Octahydro-Coumarin formed three hydrogen bonds (with Gly116, Gly117, and Ala199).

Other compounds displayed varying docking scores, indicating different binding affinities to BChE. For instance, Azelaaldehydic acid (−10.95 kJ/mol) and Nonanedioic acid (−11.36 kJ/mol) exhibited moderate binding potential, while fatty acids like Palmitic acid (−4.83 kJ/mol) and Oleic acid (−4.36 kJ/mol) showed lower scores, suggesting weaker interactions with BChE. These results highlight the significance of hydrogen bonding and specific binding interactions in determining inhibitory potency. As illustrated in Table 9 and Figure 2, these molecular docking studies provide valuable insights into the potential of peanut oil constituents as BChE inhibitors, with implications for treating Alzheimer’s and other neurodegenerative conditions.

**FIGURE 3:**
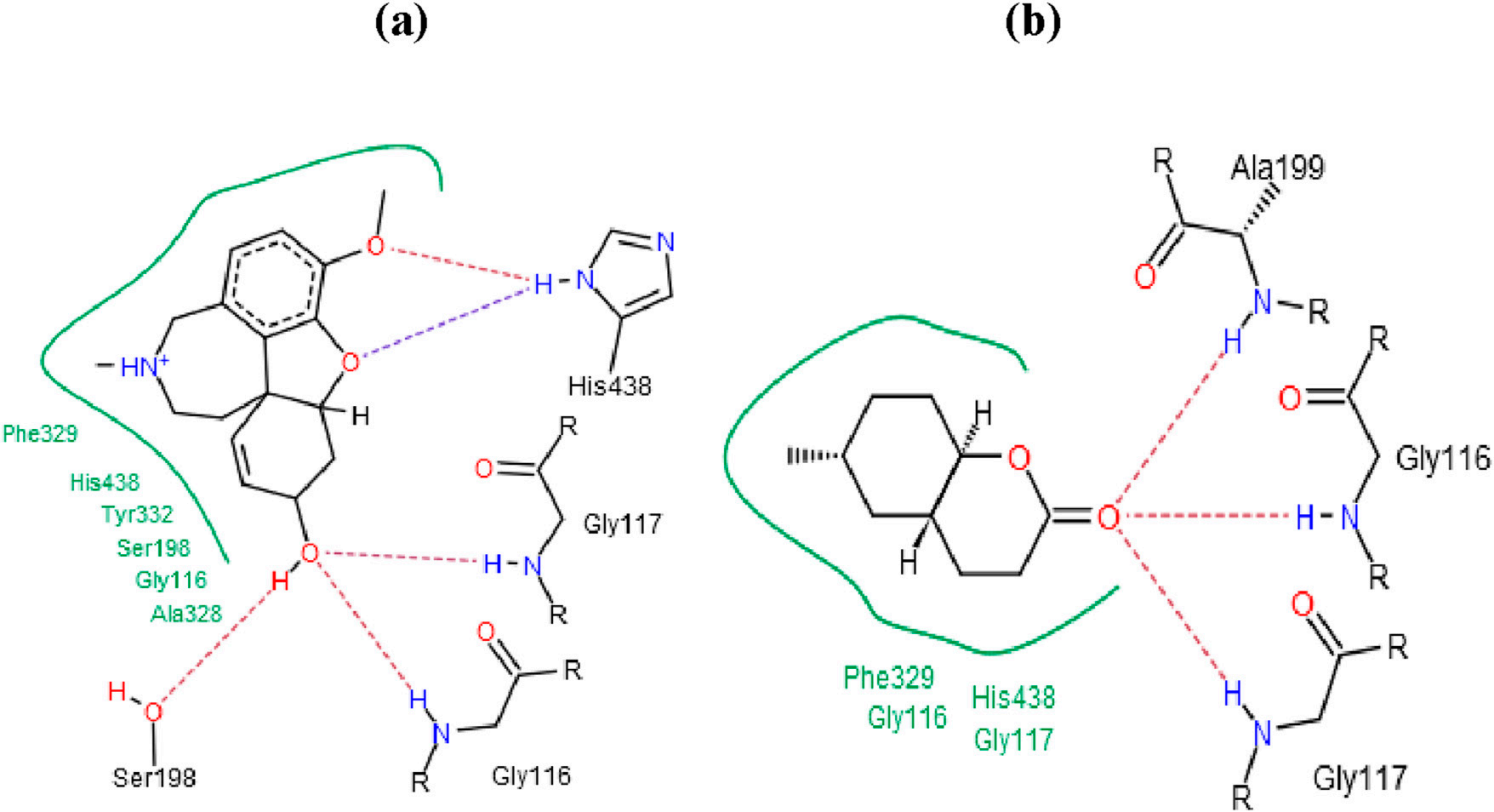
Binding Mode Interactions of Galantamine **(A)** and 6-Methyl Octahydro-Coumarin **(B)** with the BChE Active Site. This figure depicts the detailed binding interactions of Galantamine **(A)** and 6-methyl Octahydro-Coumarin **(B)** within the BChE active site. Hydrogen bonds are represented by broken lines, while the green-shaded regions indicate hydrophobic interactions. These interactions illustrate the molecular mechanisms through which each compound binds and potentially inhibits the enzyme.

Figure 3 displays the most likely binding orientations of each compound as determined by docking simulations. The catalytic anionic site (CAS) is shown in blue, while the peripheral anionic site (PAS) is in red. Ligand atoms are color-coded: carbon in green, oxygen in red, and nitrogen in blue. This positioning emphasizes key interactions within the active site that contribute to the inhibitory effects of the compounds. The difference in BChE inhibitory potency between Galantamine and 6-Methyl Octahydro-Coumarin stems from their distinct hydrogen bonding patterns. Galantamine forms five hydrogen bonds with residues, enhancing its stability and inhibitory efficacy in the BChE active site, while 6-Methyl Octahydro-Coumarin forms only three, which may explain its slightly lower inhibitory potency despite a favorable docking score. Additionally, the hydrophobic interactions, indicated in green, contribute to the binding stability and overall effectiveness of these compounds as BChE inhibitors. Specifically, 6-Methyl Octahydro-Coumarin forms three hydrogen bonds with Gly116, Gly117, and Ala199, whereas Galantamine establishes five hydrogen bonds with Ser198, Gly116, Gly117, and His438. This differential binding pattern underscores the importance of specific amino acid interactions in enhancing or limiting inhibitory efficacy (Gonzalez et al., 2016).

The low concentration of 6-Methyl Octahydro-Coumarin (1%) in the studied oil presents challenges for extraction and purification, necessitating optimization of traditional methods and a cost-effectiveness evaluation of the refining process. Although the oil is rich in polyunsaturated fatty acids (PUFAs) like oleic acid, known for their neuroprotective effects, the absence of strong BChE inhibitors likely accounts for the moderate effects observed. This aligns with findings by Lauritzen et al. (2000), which emphasize that while PUFAs can enhance cognitive health, they do not significantly inhibit cholinesterase directly. Therefore, while peanut oil may not be a potent BChE inhibitor, it could still provide neuroprotective benefits when included in the diet. Docking simulations reveal that 6-Methyl Octahydro-Coumarin has the highest binding affinity to the BChE catalytic site, yet its binding strength is much lower than that of established inhibitors like Galantamine. This suggests that while some minor components of the oil may interact with BChE, they lack the potency for significant therapeutic impact. Previous studies, such as those by (Song et al., 2019; Sakurai et al., 2021), indicate that the cognitive benefits of oleic acid may relate to its role in modulating gene expression associated with neuroinflammation and synaptic health rather than direct cholinesterase inhibition. Thus, exploring other constituents or combining peanut oil with stronger inhibitors may yield better health outcomes.

## 5 Conclusion

This study presents a pioneering investigation into the antioxidant and anti-Alzheimer’s potential of peanut oil (*A. hypogaea* L.), employing comprehensive GC-MS analysis alongside *in vitro* and *in silico* assessments. The findings reveal that peanut oil exhibits weak antioxidant activity, with a DPPH scavenging IC50 value of 1715.92 ± 93.28 μg/mL. GC-MS analysis identified oleic acid as the predominant unsaturated fatty acid, constituting 49.31% of the total fatty acids, which correlates with its antioxidant properties. In terms of anti-Alzheimer’s potential, peanut oil demonstrated moderate inhibition of butyrylcholinesterase (BChE), with an inhibition capacity of 36.47% at a concentration of 200 μg/mL. Docking simulations highlighted 6-Methyl Octahydro-Coumarin as a significant compound, achieving a docking score of −15.86 kJ/mol to BChE, though it was less potent than Galantamine, which scored −23.4 kJ/mol. The low abundance of 6-methyl octahydro-coumarin (1%) in the oil poses challenges for its extraction and purification, necessitating improvements in conventional methods. The physicochemical analysis indicated that oleic acid and palmitic acid comply with Lipinski’s rule of five, with logP values of 5.71 and 5.20, respectively, despite variability in lipophilicity and solubility. Cytotoxicity assessments demonstrated that oleic acid, palmitic acid, and stearic acid were effective against melanoma (A2058 and SK-MEL-1) and non-small cell lung cancer (NCI-H838) cell lines, while oxiraneoctanoic acid, 3-octyl, displayed significant potential against leukemia (H9). These results underscore peanut oil’s promise for antioxidant, anti-Alzheimer’s, and anticancer applications, with specific compounds emerging as candidates for further pharmaceutical development. Future research should prioritize clinical studies to explore the therapeutic potential of these compounds, particularly in treating neurodegenerative diseases and various cancers. Investigating their efficacy and safety in human populations could pave the way for significant advancements in natural treatments, potentially leading to novel and effective therapeutic approaches. Comprehensive clinical trials will be crucial for validating these promising benefits and establishing their role in medical applications, thereby enhancing the landscape of natural therapies in the fight against these diseases.

## Data Availability

The original contributions presented in the study are included in the article/Supplementary Material, further inquiries can be directed to the corresponding author.
