# Corrigendum: Antioxidant, Anti-Alzheimer’s, anticancer, and cytotoxic properties of peanut oil: *in vitro*, *in silico*, and GC-MS analysis

**DOI:** 10.3389/fchem.2025.1563210

**Published:** 2025-02-21

**Authors:** Hanène Djeghim, Djamila Benouchenne, El Hassen Mokrani, Huda Alsaeedi, David Cornu, Mikhael Bechelany, Ahmed Barhoum

**Affiliations:** ^1^ Biochemistry Laboratory, Biotechnology Research Center (CRBt), Constantine, Algeria; ^2^ Laboratoire de Génétique, Biochimie et Biotechnologie végétale, Faculté des Sciences de la Nature et de la vie, Université des Frères Mentouri Constantine 1, Constantine, Algeria; ^3^ Higher National School of Biotechnology Taoufik KHAZNADAR, nouveau Pôle universitaire Ali Mendjli, Constantine, Algeria; ^4^ Laboratory of Applied Biochemistry, Department of Biochemistry and Cellular and Molecular Biology, Faculty of Natural and Life Sciences, University Mentouri Brothers, Constantine, Algeria; ^5^ Department of Chemistry, College of Science, King Saud University, Riyadh, Saudi Arabia; ^6^ Institut Européen des Membranes, University Montpellier, ENSCM, CNRS, Montpellier, France; ^7^ Functional Materials Group, Gulf University for Science and Technology, Mubarak Al-Abdullah, Kuwait; ^8^ NanoStruc Research Group, Chemistry Department, Faculty of Science, Helwan University, Cairo, Egypt

**Keywords:** peanut oil, phytochemical profileing, antioxidant properties, anti-Alzheimer’s potential, GC-MS analysis, enzyme inhibition, docking studies, cytotoxicity assessments

In the published article, there was an error in **Affiliation 7**. Instead of “GUST, Helwan, Kuwait”, it should be “Functional Materials Group, Gulf University for Science and Technology, Mubarak Al-Abdullah, Kuwait”.

The authors apologize for this error and state that this does not change the scientific conclusions of the article in any way. The original article has been updated.

