# Supplementary material for: Antioxidant, Anti-Alzheimer’s, anticancer, and cytotoxic properties of peanut oil: in vitro, in silico, and GC-MS analysis
Source: Front Chem. 2024 Oct 24;12:1487084. doi: 10.3389/fchem.2024.1487084 (PMC11541349; doi:10.3389/fchem.2024.1487084)
Supplement: Supplementary file 1 [file Presentation1.PPTX]

## Slide 1
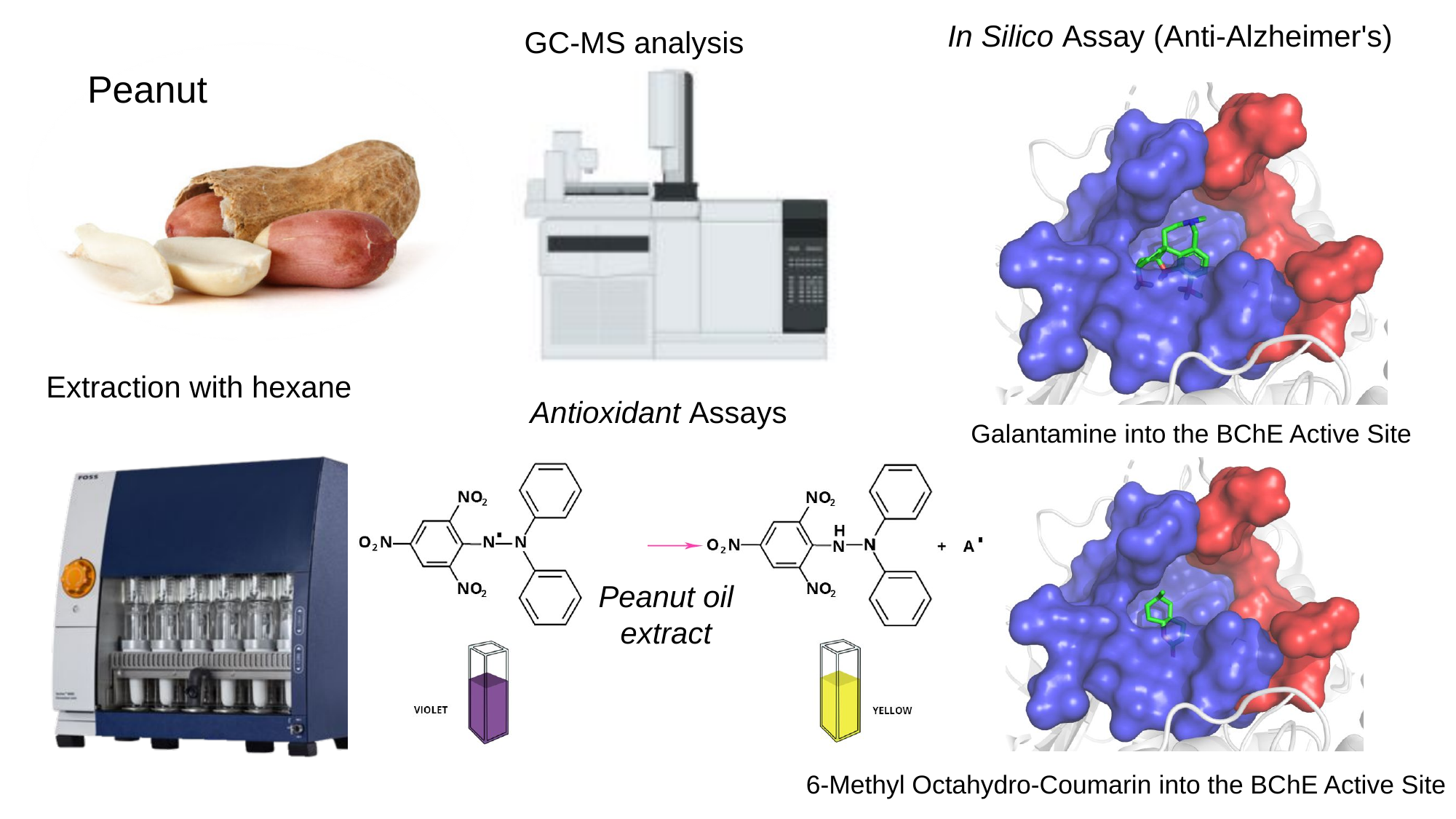

In Silico Assay (Anti-Alzheimer's)
GC-MS analysis
Peanut
Extraction with hexane
Antioxidant Assays
Galantamine into the BChE Active Site
Peanut oil
extract
6-Methyl Octahydro-Coumarin into the BChE Active Site
